# QuickStats

**Published:** 2013-05-31

**Authors:** Ellen A. Kramarow

**Figure:**
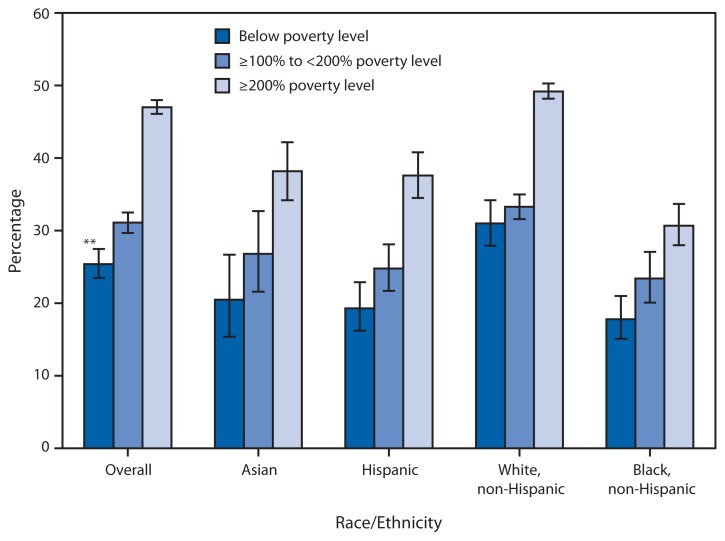
Percentage of Adults Aged ≥65 Years Who Reported Excellent or Very Good Health,^*^ by Selected Race/Ethnicity^†^ and Poverty Status^§^ — National Health Interview Survey, 2009–2011^¶^ ^*^ Respondents were asked, “Would you say your health in general is excellent, very good, good, fair, or poor?” ^†^ Persons of Hispanic ethnicity might be of any race or combination of races. ^§^ Poverty status is based on family income and family size using the U.S. Census Bureau poverty thresholds. Family income was imputed when information was missing, using multiple imputation methodology. ^¶^ Estimates are based on household interviews of a sample of the noninstitutionalized U.S. civilian population. Estimates are age adjusted using the projected 2000 U.S. population as the standard population and three age groups: 65–74 years, 75–84 years, and ≥85 years. ^**^ 95% confidence interval.

During 2009–2011, approximately 41% of adults aged ≥65 years reported their health to be excellent or very good. The percentage reporting excellent or very good health was higher among those in families with higher income compared with families with lower income. Non-Hispanic whites aged ≥65 years were more likely to report excellent or very good health at each income level compared with Asians, Hispanics, or non-Hispanic blacks.

**Sources:** National Health Interview Survey, 2009–2011. Available at http://www.cdc.gov/nchs/nhis.htm.

CDC. Health Data Interactive. Available at http://www.cdc.gov/nchs/hdi.htm.

